# Imlunestrant, an oral selective estrogen receptor degrader, in combination with HER2 directed therapy, with or without abemaciclib, in ER-positive, HER2-positive advanced breast cancer: results from the phase 1a/1b EMBER study

**DOI:** 10.1186/s13058-025-02168-6

**Published:** 2025-12-21

**Authors:** Manali Bhave, Komal L. Jhaveri, Peter A. Kaufman, Philippe Aftimos, Janine Lombard, Karthik V. Giridhar, Seock-Ah Im, Cynthia X. Ma, Kuo-Ting Lee, Sung-Bae Kim, Joohyuk Sohn, Yujia Li, Eunice Yuen, Shawn T. Estrem, Bastien Nguyen, Monish Ram Makena, Roohi Ismail-Khan, Muralidhar Beeram

**Affiliations:** 1 Emory University, Atlanta, GA USA; 2 Sloan Kettering Cancer Center, New York, NY USA; 3 Weill Cornell Medical College, New York, NY USA; 4 University of Vermont Cancer Center, Burlington, VT USA; 5 Institut Jules Bordet, Université Libre de Bruxelles, Hôpital Universitaire de Bruxelles, Brussels, Belgium; 6 Medical Oncology Department, Calvary Mater Hospital Newcastle, Newcastle, NSW Australia; 7 Department of Medical Oncology, Mayo Clinic, Rochester, MN USA; 8 Cancer Research Institute, Seoul National University Hospital, Seoul National University College of Medicine, Seoul, Republic of Korea; 9 Washington University School of Medicine, St. Louis, MO USA; 10 Department of Surgery, National Cheng Kung University Hospital, Tainan, Taiwan; 11 Asan Medical Center, University of Ulsan College of Medicine, Seoul, Republic of Korea; 12 Yonsei Cancer Center, Yonsei University College of Medicine, Seoul, Republic of Korea; 13 Eli Lilly and Company, Indianapolis, IN USA; 14 START Center for Cancer Care, San Antonio, TX USA

**Keywords:** SERD, CDK4/6 inhibitors, Breast cancer

## Abstract

**Background:**

Hormone receptor-positive (HR+), human epithelial growth factor receptor 2 (HER2) overexpressed breast cancer (BC) represents the more aggressive subtype of HR + BC, typically associated with poor clinical outcomes. Despite significant advancements in the treatment of estrogen receptor-positive (ER+)/HER2 + BC, resistance to endocrine therapy (ET) poses a continued challenge. Imlunestrant is a next-generation, oral, brain-penetrant, pure antagonistic ER degrader designed to deliver continuous ER target inhibition. It has shown favorable clinical benefit, safety, and pharmacokinetics (PK) when used as monotherapy or with targeted therapy in patients with ER+/HER2- advanced BC (ABC). Here, we present the safety and efficacy of imlunestrant with HER2 targeted therapy in patients with ER+/HER2 + ABC of the EMBER trial.

**Methods:**

Patients were randomized to imlunestrant (400 mg RP2D) + trastuzumab (group A) versus imlunestrant + trastuzumab ± abemaciclib [(150 mg twice daily) group B] or received maintenance treatment with imlunestrant + trastuzumab + pertuzumab until progression or discontinuation at standard doses (group C). In the randomized allocation, eligible patients with locally advanced or metastastic disease had received ≥ 2 prior HER2-directed regimens in the metastatic setting, and no prior treatment with CDK4/6 inhibitors or fulvestrant. The maintenance cohort (group C) was added later to include patients without disease progression after first-line induction taxane-based chemotherapy (any duration), trastuzumab, and pertuzumab. Endpoints included safety, PK, antitumor activity, and tumor biomarker assessments.

**Results:**

In total, 45 patients with ER+/HER2 + ABC were treated (group A, *n* = 18; group B, *n* = 21; group C, *n* = 6). Adverse events were consistent with the known safety profiles of the partner drugs. Imlunestrant PK was consistent with previously reported data. For groups A, B, and C, the objective response rates (ORRs) were 7%, 25%, and 33%, respectively, while the clinical benefit rates (CBRs) were 44%, 48%, and 100%. In group C, the duration of response ranged between 5.13 and 9.46 months. In groups A and B, baseline plasma ctDNA sample sequencing identified prevalent alterations in *ERBB2* amplification (57%), *CCND1* amplification (22%), and mutations in *TP53* (49%), *PIK3CA* (30%), *ESR1* (24%), and *GATA3* (14%).

**Conclusions:**

Imlunestrant in combination with trastuzumab ± abemaciclib or pertuzumab presented a safety profile that was consistent with those of the partnered drugs. No new safety findings were observed. Furthermore, imlunestrant in combination with the partnered drugs demonstrated preliminary antitumor activity in patients with ER+/HER2 + ABC.

**Trial registration:**

ClinicalTrials.gov identifier NCT04188548 (Registered 18 November 2019).

**Supplementary Information:**

The online version contains supplementary material available at 10.1186/s13058-025-02168-6.

## Introduction

Approximately 10% of breast cancers (BC) express the estrogen receptor (ER) and overexpress the human growth factor receptor 2 (HER2, also known as ERBB2) [1, 2]. ER+/HER2 + BC is more prevalent in younger patients, has distinct metastatic patterns, is more aggressive, and is associated with high mortality risk [2–4]. Approximately 50% of patients will develop brain metastasis, notably higher than with other BC subtypes [5, 6].

In HER2-positive (HER2+) advanced BC (ABC), the recommended first-line treatment is the combination of HER2-targeted therapy (trastuzumab + pertuzumab) with taxane chemotherapy (docetaxel or paclitaxel), regardless of ER status [7]. Taxanes are administered for 6–8 cycles, as tolerated, followed by maintenance therapy with pertuzumab + trastuzumab [7]. In ER+/HER2 + disease, endocrine therapy (ET) is added to the maintenance regimen and continued until progression [7].

The CLEOPATRA trial confirmed that adding pertuzumab to trastuzumab and docetaxel significantly improved overall survival in patients with HER2-positive metastatic breast cancer who had not previously received chemotherapy or anti-HER2 therapy for metastatic disease, presenting a median investigator-assessed progression-free survival of 18.7 months [8]. Of note, the CLEOPATRA trial included ER- patients while the AFT-38 PATINA trial focused on ER+/HER2 + patients.

The phase III AFT-38 PATINA trial demonstrated that adding the CDK4/6 inhibitor palbociclib into a regimen of anti-HER2 therapy (trastuzumab with or without pertuzumab) and endocrine therapy (with an aromatase inhibitor or fulvestrant) significantly extended PFS in patients with ER+, HER2 + metastatic BC following induction chemotherapy. Patients in the palbociclib treatment arm had a median PFS of 44 months, compared to 29 months for those treated with anti-HER2 therapy and endocrine therapy alone, representing a statistically significant improvement (*P* = 0.0074). This 15-month extension in disease control represented a practice changing benefit, supporting the use of palbociclib and potentially other CDK4/6 inhibitors in this patient population [9].

Despite these advances, eventual treatment resistance remains a challenge, ultimately leading to disease progression [10, 11]. This highlights the need for novel therapeutic strategies, potentially involving the incorporation of a more optimized endocrine treatment backbone. The co-expression of ER and HER2 affects the response to HER2 targeted therapy and ET due to pathway crosstalk. Blocking both ER and HER2 may overcome resistance [3]. Combining fulvestrant, a first-generation selective estrogen receptor degrader (SERD) that inhibits estrogen signaling, with trastuzumab resulted in a median progression-free survival (PFS) of 6.4 months (95% confidence interval [CI], 3.5–8.2) and a median overall survival of 35.3 months (95% CI, 20.0–46.7) after standard HER2-targeted therapy in patients with ER+/HER2 + ABC [12]. This provided a rationale for evaluating adding oral SERDs to HER2-targeted therapy.

In the phase 2 NA-PHER2 study, combining fulvestrant, pertuzumab, trastuzumab, and palbociclib (a cyclin-dependent kinase 4 and 6 [CDK4/6] inhibitor) achieved a 97% objective response rate (ORR) in the neoadjuvant setting [13]. Similarly, the phase 2 monarcHER study showed that the combination of fulvestrant + trastuzumab + abemaciclib significantly improved PFS to 8.3 months compared to 5.7 months with standard chemotherapy + trastuzumab [14]. Despite its benefits, fulvestrant’s painful intramuscular administration, burdensome monthly administration schedule, and inability to deliver sustained ER inhibition throughout the dosing interval limits its usefulness and application [15–17].

Imlunestrant is a next-generation, novel, oral SERD, and a pure ER antagonist [18, 19]. Preclinical studies demonstrated imlunestran had antitumor activity in both *ESR1* wild-type and mutant models. Imlunestrant can cross the blood-brain barrier and improved survival in a brain orthotopic mouse model [19]. In the phase 1 EMBER study, imlunestrant, as monotherapy and combined with targeted therapy, demonstrated a favorable pharmacokinetic (PK) and safety profile with encouraging preliminary antitumor efficacy in patients with ER+/HER2-negative (HER2-) ABC [18].

In this report, we present data from the EMBER study on the combination of imlunestrant with HER2-targeted therapy in patients with ER+/HER2 + ABC.

## Methods

### Study design

EMBER (NCT04188548) is a global, open-label trial that began with a phase 1a dose-escalation of imlunestrant monotherapy followed by phase 1b with multiple dose-expansion cohorts evaluating imlunestrant monotherapy and in combination with targeted therapy. The study included patients with ER+ (HER2- and HER2+) ABC and recurrent, persistent, or metastatic ER + endometrial endometrioid cancer (EEC). Recruitment occurred in 76 centers across 8 countries from December 2019 to March 2023.

**Fig. 1:**
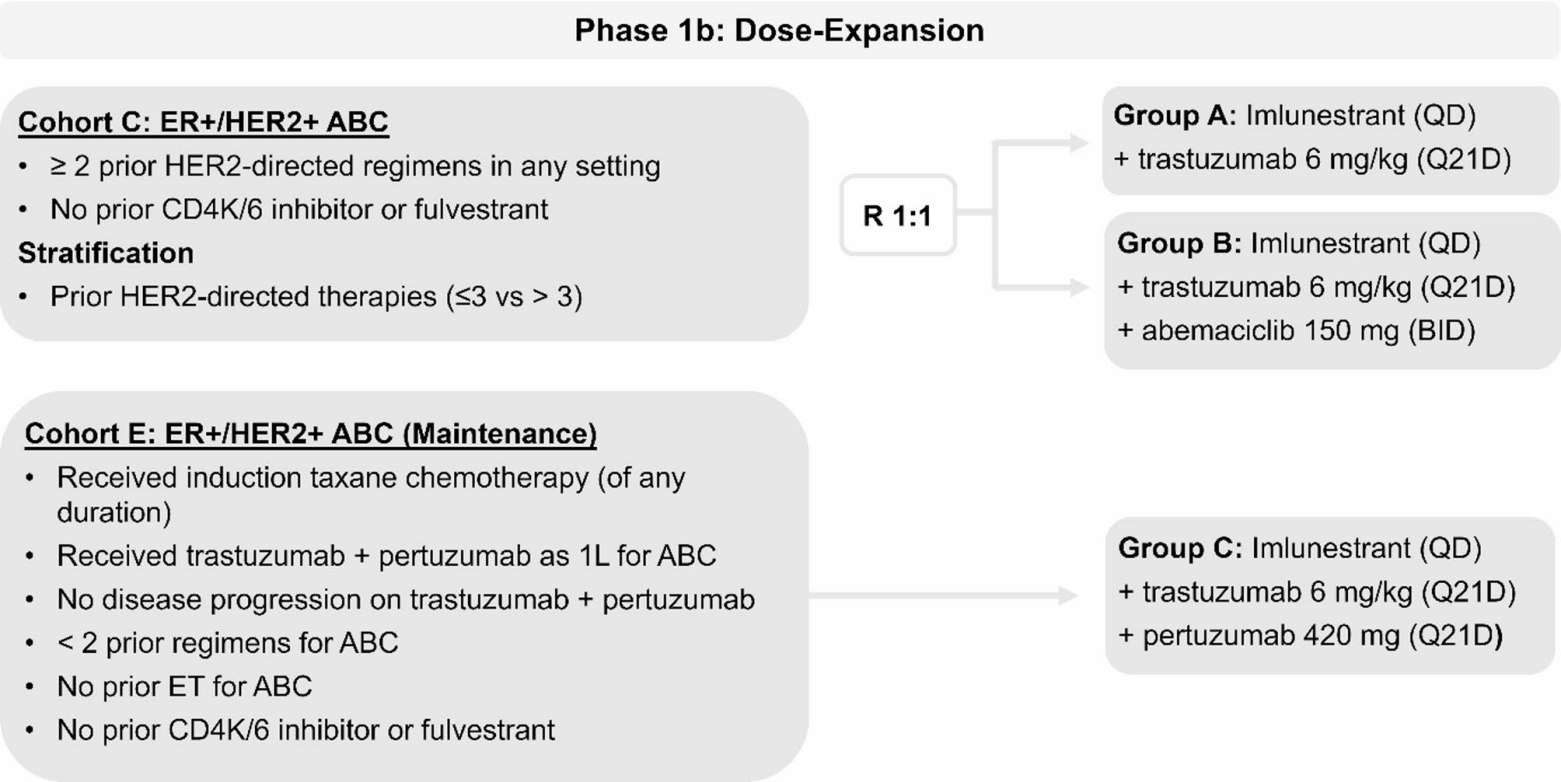
Study Design. Abbreviations: ABC, advanced breast cancer; CDK4/6, cyclin-dependent kinase 4/6; ER, estrogen receptor; ET, endocrine therapy; HER2, human epidermal growth factor receptor 2; QD, once daily; Q21D, every 21 days

**Fig. 2:**
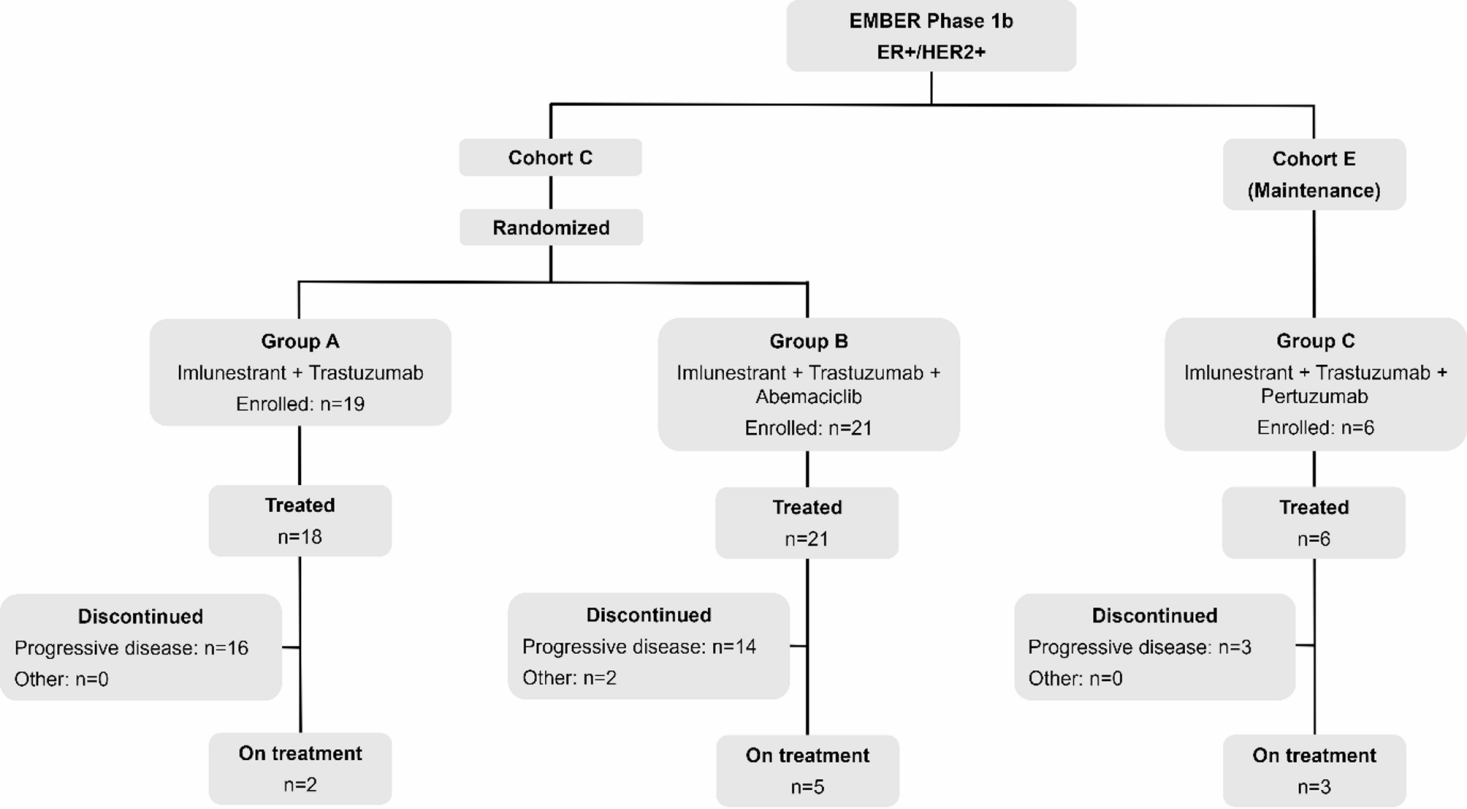
CONSORT diagram. Abbreviations: ER+, estrogen receptor-positive; HER2, human epidermal growth factor receptor 2-positive

In phase 1a, imlunestrant was administered in dosing cohorts ranging from 200 to 1200 mg to determine the recommended phase 2 dose (RP2D) using the i3 + 3 design [20]. Detailed study design and results for other cohorts have been previously reported [18]. This report focuses on cohorts C and E (Figs. 1 and 2).

Cohort C included patients with ER+/HER2 + ABC who were randomized to receive imlunestrant + trastuzumab (group A) and imlunestrant + trastuzumab + abemaciclib (group B).

Cohort E was added later and focused on maintenance patients who received imlunestrant + trastuzumab + pertuzumab (group C). Cohort E is referred to as the maintenance cohort as ET is often combined with maintenance trastuzumab + pertuzumab after completion of first-line induction chemotherapy for ER+/HER2 + ABC [21].

### Endpoints

The primary objective of the overall EMBER study was RP2D determination in patients with HER2- BC and EEC and was reached prior to enrollment in the HER2 + cohorts [18, 22]. The RP2D for HER2- and EEC was subsequently used in the HER2 + cohorts. Secondary objectives included assessment of safety, tolerability, pharmacokinetics, clinical benefit rate (CBR), disease control rate (DCR), PFS, and ORR, per Response Evaluation Criteria in Solid Tumors v1.1. Exploratory objectives included tumor biomarker assessments. The data cutoff date was August 14, 2023.

### Patients

Eligible patients with ER+/HER2 + BC with locally advanced unresectable or metastatic disease were enrolled. ER + was defined as ≥ 1% ER + tumor nuclei by immunohistochemistry [23]. HER2 + status was determined by in situ hybridization, fluorescence in situ hybridization, or immunohistochemistry methodologies per 2018 ASCO/CAP HER2 guidelines [24]. For cohort C, groups A and B (imlunestrant + trastuzumab ± abemaciclib), patients must have received ≥ 2 prior HER2-directed regimens and no prior CDK4/6 inhibitors or fulvestrant.

For the maintenance cohort (cohort E, group C: imlunestrant + trastuzumab + pertuzumab), patients had completed induction taxane-based chemotherapy (4–6 cycles) + trastuzumab + pertuzumab as first-line therapy for ABC. While the standard induction period lasts 4–6 cycles (approximately 3–6 months of therapy), we were not able to determine how many cycles each patient had received but only that they had completed induction therapy. Disease progression while on trastuzumab + pertuzumab maintenance therapy was not allowed. Patients in the maintenance cohort must have received no more than one prior ABC regimen, had no history ET for ABC, and no previous CDK4/6 inhibitors.

Pre-menopausal women received concomitant gonadotropin-releasing hormone agonists. An Eastern Cooperative Oncology Group performance status (ECOG PS) of 0–1 and normal cardiac function with a left ventricular ejection fraction of ≥ 50% at baseline (determined by echocardiography or multigated acquisition) was required.

Key exclusion criteria included systemic disorders (e.g., HIV, active hepatitis B/C, renal impairment, interstitial lung disease, cardiac conditions), visceral crisis, inflammatory BC, or symptomatic central nervous system (CNS) metastasis and/or carcinomatous meningitis. Patients with treated CNS metastasis were considered eligible if they had not received corticosteroids and/or anticonvulsant treatment at least 14 days prior to first dose of study treatment. Eligible patients with CNS metastasis were required to present asymptomatic disease and have radiographical stability confirmed by repeat imaging at least 30 days prior to providing consent.

### Study procedures

Imlunestrant was administered once daily (QD) on an empty stomach as capsules or tablets. Targeted therapy in the combination regimens was administered according to their respective labels. Imlunestrant omissions < 28 days and dose reductions were allowed as per protocol unless patients were already at the lowest dose (200 mg). Dose modifications for partner agents were determined by the investigator in accordance with the approved product labels. Study treatment continued until disease progression, unacceptable toxicity, or patient withdrawal.

### Assessments

Study visits occurred weekly in the first month (phase 1a only) and then monthly. Visits included physical, laboratory, cardiological, and ECOG PS assessments. Follow-up visits occurred approximately 30 days after treatment discontinuation, followed by long-term survival monitoring every 12 weeks (clinic/phone visit). Tumor assessment occurred at baseline, then every 8 weeks for the first 6 months, and then every 12 weeks until study discontinuation. ECHO/MUGA were repeated every 3 months (± 7 days) during treatment per trastuzumab label.

Blood samples for PK were collected on Cycle (C) 1 Day (D) 1, C2D1 and C3D1 to assess imlunestrant plasma concentrations using liquid chromatography-mass spectrometry (LC-MS/MS) methods. Pharmacokinetic parameters were calculated using standard noncompartmental methods.

Safety was evaluated using the National Cancer Institute Common Terminology Criteria for Adverse Events (AEs) v5.0 and coded using the Medical Dictionary for Regulatory Activities.

Pre-treatment samples for plasma biomarker analysis were collected on C1D1 in Cell-Free DNA BCT (Streck, La Vista, NE) for circulating tumor DNA (ctDNA) analyses. The Guardant360 assay (Guardant Health, Redwood City, CA) was used to perform ctDNA somatic mutation analysis. Mutation analyses included only somatic variants known or predicted to have oncogenic activity.

### Statistical analysis

The safety population included patients who received ≥ 1 dose of imlunestrant. ORR (defined as the proportion of patients with the best overall response [BOR] of complete response [CR] and partial response [PR]) was evaluated in patients with measurable disease. CBR (defined as the proportion of patients with a BOR of CR, PR, or stable disease for ≥ 24 weeks), DCR (defined as the proportion of patients with a BOR of CR, PR or stable disease), DOR (defined as the time from the date measurement criteria for CR or PR [whichever is first recorded] are first met until the first date that disease recurrence or progression is observed) and PFS were analyzed in the safety population. Curves were estimated by the Kaplan-Meier method. Median and 95% CIs were calculated by log-log method. The analyses were conducted by cohorts so that patients with different treatments were analyzed separately. The data cutoff date for the analysis was August 14, 2023.

## Results

### Patient baseline characteristics

From December 2019 to August 2023, a total of 307 patients with ER+ (HER2- or HER2+) ABC were treated with imlunestrant monotherapy (*n* = 262) or with other agents (*n* = 45). Among these, 45 with ER+/HER2 + ABC were treated with imlunestrant in combination with targeted therapy: imlunestrant + trastuzumab (*n* = 18; group A), imlunestrant + trastuzumab + abemaciclib (*n* = 21; group B), and imlunestrant + trastuzumab + pertuzumab (*n* = 6; group C). In the neoadjuvant/adjuvant EBC setting, prior HER2-directed therapy was administered to 39% (*n* = 7), 24% (*n* = 5), 33% (*n* = 2) of patients in groups A, B and C, respectively. As expected, in the advanced setting, 100% in each group received prior HER2-directed therapy. At baseline, 82% (*n* = 37) across all groups had measurable disease (Table 1).

**Table 1:** Baseline demographics and disease characteristics

	Imlunestrant + trastuzumab	Imlunestrant + trastuzumab + abemaciclib	Imlunestrant + trastuzumab + pertuzumab
	*n* = 18	*n* = 21 ^a^	*n* = 6
Median age, years (range)	61 (37–81)	54 (32–74)	49 (31–67)
Race, n (%)			
Asian	7 (39)	11 (52)	0
Black or African American	3 (17)	0	0
White	8 (44)	10 (48)	6 (100)
Menopausal status, n (%)			
Postmenopausal	16 (89)	17 (81)	3 (50)
Pre-menopausal	2 (11)	4 (19)	3 (50)
Baseline ECOG PS, n (%)			
0	14 (78)	13 (62)	5 (83)
1	4 (22)	8 (38)	1 (17)
ER+, HER2 + per local assessment, n (%)^b^	18 (100)	21 (100)	6 (100)
*ESR1* mutations detected at baseline, n/N (%) ^c^	4/17 (24)	5/20 (25)	0
*PIK3CA* mutations detected at baseline, n/N (%) ^c^	4/16 (25)	6/20 (30)	0
PgR-, n (%)	4 (22)	5 (24)	1 (17)
Visceral metastasis, n (%)	9 (50)	12 (57)	4 (67)
Measurable disease at baseline, n (%)	14 (78)	20 (95)	3 (50)
No. of prior therapies any setting, median (range)	5 (3–10)	4 (2–8)	2 (1–3)
No. of prior therapies for ABC, median (range)	4 (1–10)	3 (1–7)	1 (1–1)
Prior HER2-directed therapy, n (%)	18 (100)	21 (100)	6 (100)
(Neo) adjuvant	7 (39)	5 (24)	2 (33)
Trastuzumab	7 (39)	5 (24)	2 (33)
Pertuzumab	3 (17)	0	0
Advanced setting	18 (100)	21 (100)	6 (100)
Trastuzumab	16 (89)	19 (91)	6 (100)
Pertuzumab	16 (89)	15 (71)	6 (100)
Trastuzumab emtansine	15 (83)	20 (95)	0
Lapatinib	4 (22)	6 (29)	0
Trastuzumab deruxtecan	6 (33)	5 (24)	0
Prior endocrine therapy, n (%)			
(Neo) adjuvant	7(39)	9 (43)	2 (33)
Advanced setting	10 (56)	12 (57)	0
Prior chemotherapy, n (%)			
(Neo) adjuvant	8 (44)	10 (48)	1 (17)
Advanced setting	17 (94)	20 (95)	6 (100)

Imlunestrant was administered at the recommended phase 2 dose of monotherapy unless otherwise stated. ^a^ One patient had received 800mg; ^b^ Per local assessment; ^c^Per central assessment of ctDNA

Abbreviations: ABC, advanced breast cancer; ctDNA, circulating tumor DNA; ECOG PS, Eastern Cooperative Oncology Group performance status; ER, estrogen receptor; HER2, human epidermal growth factor receptor 2; n, number of patients; N, number of patients with available ctDNA data; RP2D, recommended phase 2 dose

At the data cutoff (August 2023), 2 (11%), 5 (24%) and 3 (50%) patients remained on treatment with group A, group B, and group C, respectively (Fig. 2). Most discontinued due to progressive disease: 16 of 18 (84%) in group A, 14 of 21 (67%) in Group B, and 3 of 6 (50%) in group C. Additionally, in group B, 1 withdrew and another was discontinued due to physician decision. The median follow-up time was 5.8, 4.2, and 11.3 months in the cohorts treated in Group A, Group B, or Group C, respectively.

### Safety

The safety population included 45 patients who initiated treatment, 18 in group A, 21 in group B, and 6 in group C (Table 2). Approximately 98% experienced at least one treatment-emergent AE (TEAE) and the most common TEAEs include diarrhea (58%), fatigue (42%) and nausea (36%). The reported TEAEs were primarily grade 1–2. SAEs were reported in 8 (18%) patients (Group A: 4 (22%); Group B: 4 (19%); Group C: 0 (0%), with 3 related to study treatment (Table S1). There were no SAEs in Group C. There were no deaths due to AEs. Eight patients had an imlunestrant or abemaciclib dose reduction due to TEAEs. TEAEs and TRAEs are detailed in Table 3.

**Table 2:** Overall summary of AEs

	Imlunestrant + trastuzumab (Group A)	Imlunestrant + trastuzumab + abemaciclib (Group B)	Imlunestrant + trastuzumab + pertuzumab (Group C)
Parameter, n (%)	*n* = 18	*n* = 21​	*n* = 6
Patients with ≥ 1 TEAE​	17 (94)	21 (100)	6 (100)
Related to study treatment​ ^a^	13 (72)	21 (100)	4 (67)
Patients with ≥ 1 Grade ≥ 3 TEAE​	6 (33)	11 (52)	1 (17)
Related to study treatment​ ^a^	1 (6)	9 (43)	0
Patients with ≥ 1 SAE​	4 (22)	4 (19)	0
Related to study treatment	1 (6)	2 (10)	0
Study discontinuation due to AE​	0	0	0
Related to study treatment​	0	0	0
Deaths due to AEs	0	0	0
Patients with dose omissions due to TEAEs ^a^	3 (17)	11 (52)	0
Patients with dose reductions due to TEAEs^a^	0	8 (38)	0

AE, adverse event; SAE, serious adverse event; TEAE, treatment-emergent adverse event; TRAE, treatment-related adverse event. ^a^ Related to any treatment drug

**Table 3:** Incidence of TEAEs and TRAEs occurring in ≥ 10% of patients

	TEAEs							TRAEs					
	Imlunestrant + trastuzumab		Imlunestrant + trastuzumab + abemaciclib		Imlunestrant + trastuzumab + pertuzumab			Imlunestrant + trastuzumab		Imlunestrant + trastuzumab + abemaciclib		Imlunestrant + trastuzumab + pertuzumab	
Parameters, n (%)	*n* = 18		*n* = 21 ^a^		*n* = 6			*n* = 18		*n* = 21 ^a^		*n* = 6	
Patients with ≥ 1 TEAE	All	G ≥ 3	All	G ≥ 3	All	G ≥ 3		All	G ≥ 3	All	G ≥ 3	All	G ≥ 3
	17 (94)	6 (33)	21 (100)	11 (52)	6 (100)	1 (1.7)		13 (72)	1 (6)	21 (100)	9 (43)	4 (67)	0
Gastrointestinal disorders													
Nausea	5 (28)	0	10 (48)	0	1 (17)	0		5 (28)	0	10 (48)	0	0	0
Constipation	4 (22)	0	2 (10)	0	0	0		1 (6)	0	0	0	0	0
Diarrhea	2 (11)	0	21 (100)	4 (19)	3 (50)	0		2 (11)	0	21 (100)	4 (19)	1 (17)	0
Abdominal pain	2 (11)	0	7 (33)	1 (5)	0	0		0	0	5 (24)	0	0	0
Vomiting	1 (6)	0	7 (33)	0	2 (33)	0		0	0	6 (29)	0	0	0
Stomatitis	1 (6)	0	4 (19)	0	2 (33)	0		1 (6)	0	3 (14)	0	1 (17)	0
Flatulence	0	0	2 (10)	0	0	0		0	0	1 (5)	0	0	0
Blood and lymphatic system disorders													
Anemia	4 (22)	0	8 (38)	0	1 (17)	0		2 (11)	0	6 (29)	0	0	0
Hypokalemia	1 (6)	0	4 (19)	1 (5)	0	0		0	0	2 (10)	0	0	0
Neutropenia^b^	1 (6)	1 (6)	12 (57)	5 (24)	0	0		1 (6)	1 (6)	10 (48)	5 (24)	0	0
Thrombocytopenia^c^	0	0	8 (38)	4 (19)	0	0		0	0	6 (29)	3 (14)	0	0
Leukopenia	0	0	3 (14)	0	0	0		0	0	3 (14)	0	0	0
Hyponatremia	0	0	2 (10)	0	0	0		0	0	1 (5)	0	0	0
Liver disorders													
ALT increased	2 (11)	0	2 (10)	0	0	0		1 (6)	0	2 (10)	0	0	0
AST increased	1 (6)	0	3 (14)	1 (5)	0	0		0	0	1 (5)	1 (5)	0	0
Renal and urinary disorders													
Urinary tract infection	0	0	2 (10)	0	1 (17)	0		0	0	0	0	0	0
Blood creatinine increased	0	0	6 (29)	0	0	0		0	0	5 (24)	0	0	0
Respiratory, thoracic and mediastinal disorders													
Pleural effusion	2 (11)	2 (11)	0	0	0	0		0	0	0	0	0	0
COVID-19	2 (11)	0	3 (14)	0	2 (33)	0		0	0	0	0	0	0
Dyspnea	1 (6)	0	2 (10)	0	1 (17)	0		0	0	0	0	0	0
Cough	0	0	2 (10)	0	1 (17)	0		0	0	1 (5)	0	0	0
Pneumonitis	0	0	2 (10)	2 (10)	0	0		0	0	2 (10)	2 (10)	0	0
Pain													
Back pain	1 (6)	0	1 (5)	0	1 (17)	0		0	0	0	0	0	0
Arthralgia	0	0	3 (14)	0	3 (50)	0		0	0	0	0	0	0
Myalgia	0	0	3 (14)	0	1 (17)	0		0	0	1 (5)	0	1 (17)	0
Pain in extremity	1 (6)	0	3 (14)	0	0	0		1 (6)	0	0	0	0	0
Skin and subcutaneous disorders													
Alopecia	2 (11)	0	3 (14)	0	1 (17)	0		2 (11)	0	2 (10)	0	0	0
Rash^d^	1 (6)	0	5 (24)	0	2 (33)	0		1 (6)	0	3 (14)	0	0	0
Pruritus	1 (6)	0	2 (10)	0	1 (17)	0		0	0	2 (10)	0	0	0
Brain disorders													
Headache	0	0	6 (29)	0	1 (17)	0		0	0	1 (5)	0	0	0
Dizziness	0	0	3 (14)	0	2 (33)	0		0	0	1 (5)	0	0	0
Others													
Fatigue ^e^	6 (33)	0	11 (52)	0	2 (33)	0		5 (28)	0	10 (48)	0	2 (33)	0
Peripheral sensory neuropathy	2 (11)	0	0	0	2 (33)	0		0	0	0	0	1 (17)	0
Decreased appetite	0	0	7 (33)	1 (5)	0	0		0	0	5 (24)	1 (5)	0	0
Pyrexia	0	0	4 (19)	0	0	0		0	0	2 (10)	0	0	0
Hot flashes	0	0	2 (10)	0	1 (17)	0		0	0	2 (10)	0	0	0
Epistaxis	0	0	2 (10)	0	0	0		0	0	1 (5)	0	0	0
Neuropathy peripheral	0	0	2 (10)	0	0	0		0	0	0	0	1 (17)	0
Edema peripheral	0	0	2 (10)	0	2 (33)	0		0	0	0	0	0	0

ALT, alanine transminase; AST; aspartate aminotransferase; TEAE, treatment-emergent adverse event; treatment-related adverse event

^a^ One patient received 800 mg of imlunestrant

^b^ The group term of neutropenia includes neutropenia and neutrophil count decreased

^c^The group term of thrombocytopenia include thrombocytopenia and platelet count decreased

^d^The group term of rash includes rash, rash vesicular, rash maculopapular, rash morbilliform, rash pruritic, rash pustular, rash erythematous, and dermatitis acneiform

The group term of fatigue includes fatigue, and asthenia

### Group A: Imlunestrant + Trastuzumab

Seventeen (94%) patients experienced at least 1 TEAE. The most common any-grade events were fatigue (*n* = 6, 33%), nausea (*n* = 5, 28%) and anemia (*n* = 4, 22%). Grade ≥ 3 events were observed in 33% of patients, with pleural effusion reported in 2 (11%).

The most frequently reported all grade TRAEs were fatigue and nausea (*n* = 5, 28%, respectively). One patient experienced a grade ≥ 3 TRAE of neutropenia (6%). SAEs were reported in 22% (*n* = 4), with one related to treatment (**Table ****S1**).

There were no dose reductions or discontinuations due to AEs. Dose omissions due to AEs occurred in 1 (6%) patient.

### Group B: Imlunestrant + Trastuzumab + Abemaciclib

All 21 patients experienced at least one TEAE. The most frequent TEAEs were diarrhea (*n* = 21, 100%), neutropenia (*n* = 12, 57%), fatigue (*n* = 11, 52%), and nausea (48%, *n* = 10). These were also the most frequently reported TRAEs. The most frequent grade ≥ 3 TEAEs were neutropenia (*n* = 5, 24%), diarrhea (*n* = 4, 19%), and thrombocytopenia (*n* = 4, 19%). Neutropenia (*n* = 5, 24%) and diarrhea (*n* = 4, 19%) were also the most frequent grade ≥ 3 TRAEs. Additionally, 19% of patients experienced SAEs, of which 10% were related to the treatment (Table **S1**).

Eight patients (38%) had a dose reduction due to TEAE. Abemaciclib was reduced in 7 (33%) patients (due to diarrhea, neutropenia, decreased appetite, and decreased platelet count). One patient had dose reductions of both abemaciclib and imlunestrant due to grade 3 diarrhea. One discontinued only abemaciclib due to diarrhea. Dose omissions due to TEAE were observed in 13 patients (62%) including 10 (48%) with imlunestrant and abemaciclib omission and 3 (14%) with abemaciclib omission. The AEs leading to the most frequent dose omissions diarrhea (*n* = 8, 38%), neutrophil count decreased (*n* = 3, 14%), COVID-19 (*n* = 2, 10%) and platelet count decrease (*n* = 2, 10%).

### Group C: Imlunestrant + Trastuzumab + Pertuzumab

Among the 6 patients in group C, the most frequent all grade TEAEs were diarrhea and arthralgia (*n* = 3, 50% each) (Table 3). One grade ≥ 3 event, squamous cell carcinoma, was observed (17%).

The most common all grade TRAE was fatigue (*n* = 2, 33%). Importantly, there were no grade ≥ 3 events, SAEs, dose reductions, discontinuations or dose omissions due to AEs in this Cohort.

### Pharmacokinetics

**Fig. 3:**
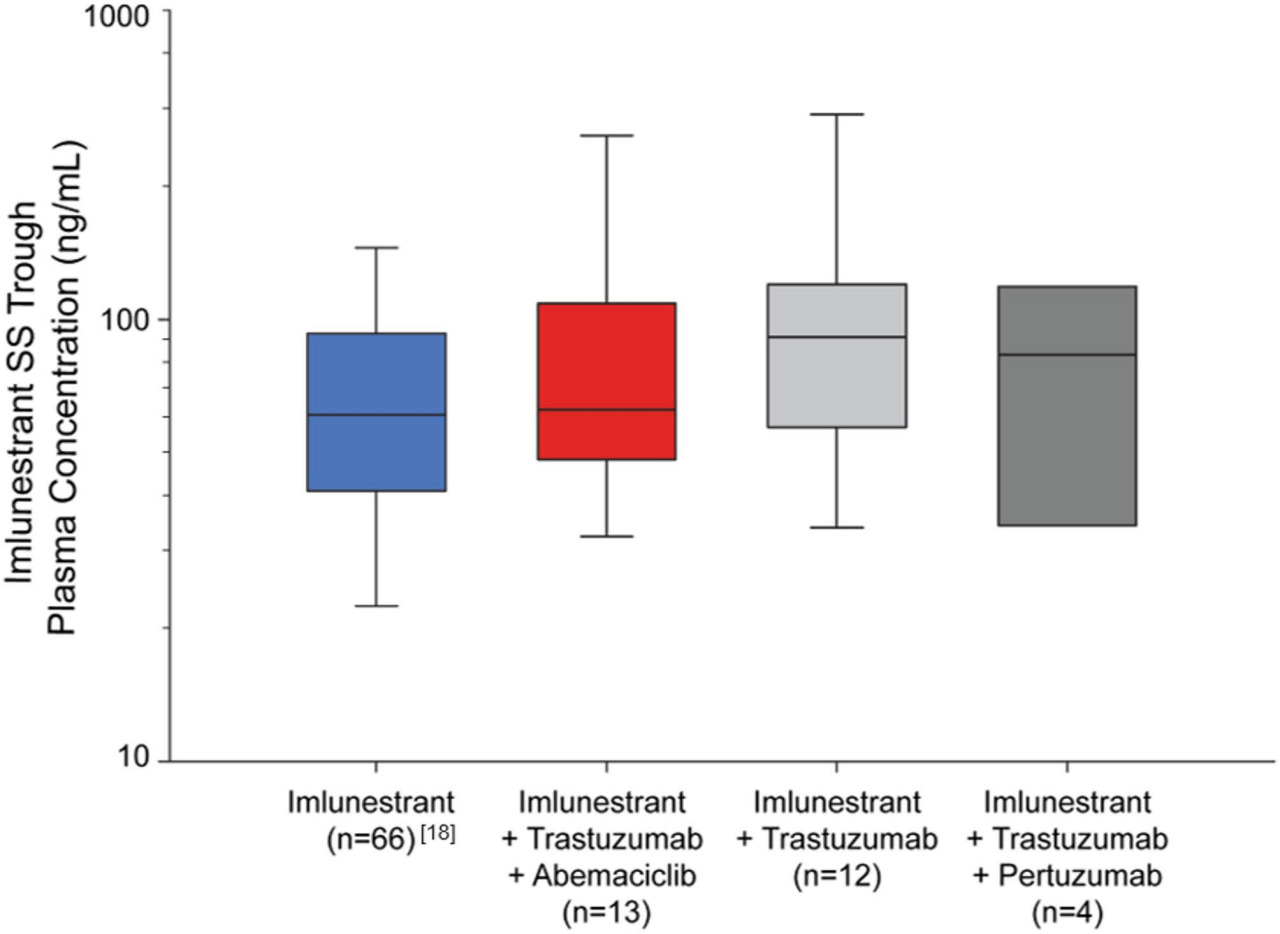
Average imlunestrant trough concentrations in plasma at steady-state across different cohorts. The box plot represents the 25th, 50th, and 75th percentiles of imlunestrant concentrations, and the whiskers represent the 10th and 90th imlunestrant percentiles. Imlunestrant monotherapy reference values as previously presented in [18]

Imlunestrant PK have been previously described [18]. Steady-state trough imlunestrant concentrations in patients receiving combination therapy were similar to those previously described (Fig. 3).

### Efficacy

**Fig. 4:**
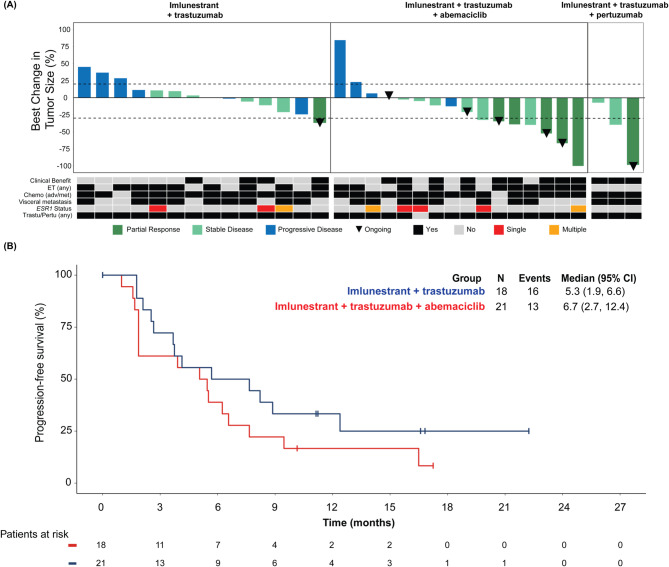
Tumor response in patients with ER+/HER2 + ABC who received imlunestrant in combination with targeted therapy. **A** Waterfall plot for best percentage change in tumor size across patients with measurable ABC who received imlunestrant + trastuzumab, imlunestrant + trastuzumab + abemaciclib, and imlunestrant + trastuzumab + pertuzumab. Each bar represents one patient. **B** Kaplan-Meier estimates of PFS. Abbreviations: ABC, advanced breast cancer; CI, confidence interval; ET, endocrine therapy; PFS, progression-free survival

In group A, CBR was 44%, DCR was 61%, median PFS was 5.3 months (95% CI, 1.9, 6.6) and ORR was 7% (Fig. 4; Table S2). In group B, CBR was 48%, DCR was 62%, median PFS was 6.7 months (95% CI, 2.7, 12.4), and ORR was 25%. Among patients from group C, both the CBR and DCR were 100%, median PFS in the maintenance portion was 15.8 (8.3, NE) and ORR was 33% (Fig. 4; Table S2). Duration of response for patients in group C ranged from 5.13 to 9.46 months.

### CtDNA

**Fig. 5:**
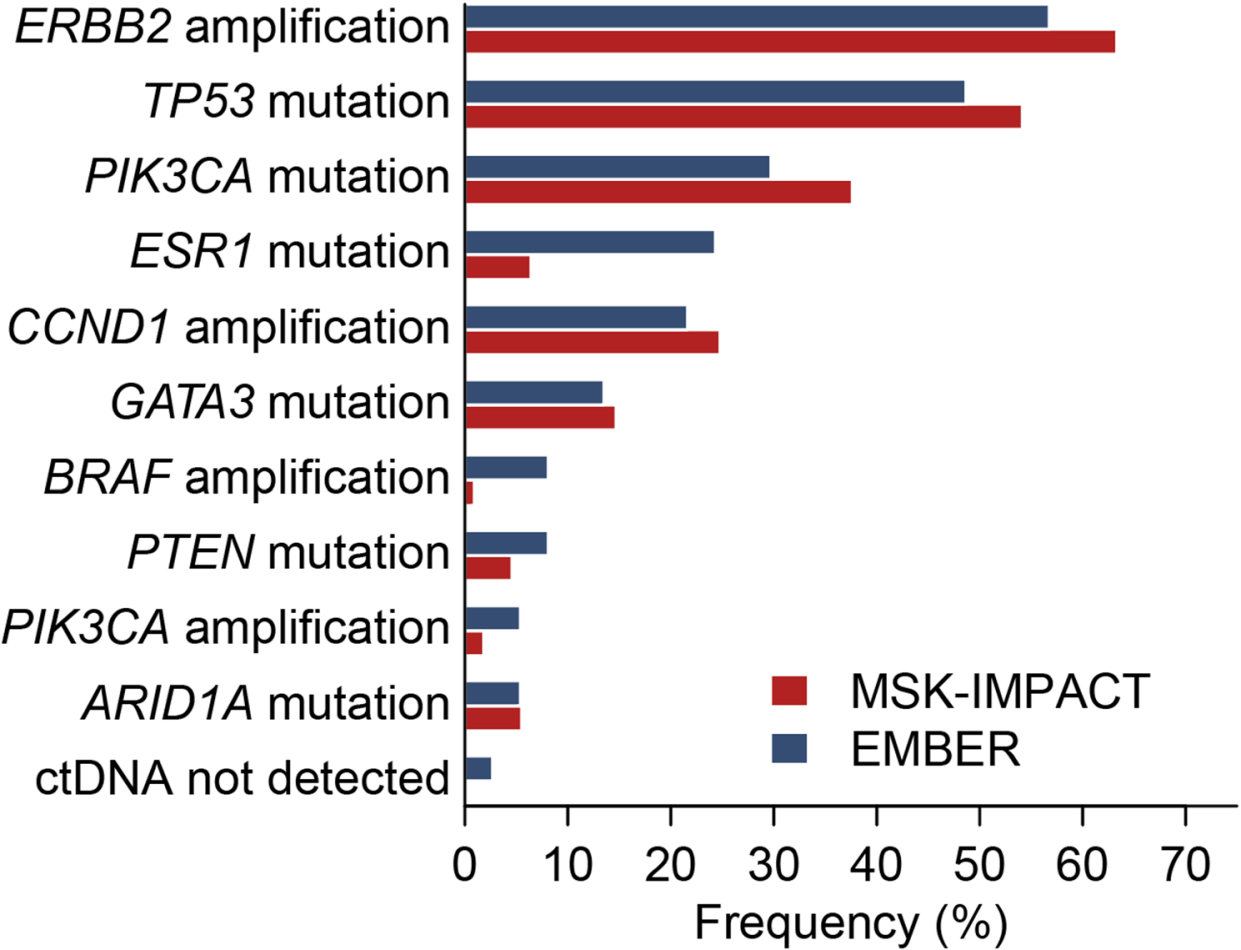
Baseline ctDNA estimates in pre-treated ER + HER2 + ABC. All EMBER patients were treated with a combination of imlunestrant + trastuzumab ± abemaciclib (*n* = 37)

Of the 39 patients in group A and group B, 37 had baseline plasma samples successfully sequenced and 36 had detectable ctDNA. All of the 6 patients in Group C had baseline plasma samples. ctDNA was only detected in 2 patients and not analyzed. The most frequently detected gene amplifications were *ERBB2* (57%) and *CCND1* (22%). The most frequent genes with detected mutation prevalence were *TP53* (49%), *PIK3CA* (30%), *ESR1* (24%), and *GATA3* (14%) (Fig. 5).

## Discussion

This report presents the results from the phase 1 EMBER study, focusing on patients with ER+/HER2 + ABC and assessing the safety and pharmacokinetics of imlunestrant as monotherapy and in combination with other approved standard of care treatment options. Specifically, the cohorts presented here evaluated the efficacy of imlunestrant in combination with HER2-targeted agents in heavily pre-treated patients who were CDK4/6 inhibitor naïve. To evaluate safety and signals of efficacy in patients having achieved a maximum response to first-line taxane chemotherapy, imlunestrant was added to HER2-directed therapy in a maintenance cohort. This approach also enabled the evaluation of imlunestrant as a potentially more optimized endocrine treatment backbone, compared to non-steroidal AIs or fulvestrant.

The safety findings presented for patients with ER+/HER2 + ABC, are consistent with the previously reported safety profile for imlunestrant in patients with ER+/HER2- ABC and those of the partner drugs [18]. The AEs observed were generally low-grade and manageable (Table 3). These data suggest that imlunestrant does not exacerbate AEs associated with trastuzumab, abemaciclib, and/or pertuzumab [10, 25, 26]. As such, imlunestrant may represent an alternative ET and could be further evaluated as a more optimized ET backbone in combination with HER2-directed therapy for advanced ER+/HER2 + BC.

To further support its potential as an optimized endocrine backbone, it is important to consider the mechanism of action of SERDs such as imlunestrant. SERDs specifically target the ERα and induce degradation by forming an unstable protein complex. SERDs can overcome resistance to aromatase inhibitors and selective ER modulators [27]. Although the first-generation SERD, fulvestrant, delivered PFS benefits in ER + ABC, it has notable limitations. Due to poor solubility, fulvestrant requires intramuscular administration [28] which is painful, negatively impacts patients’ quality of life, and does not sustain ER inhibition throughout the monthly dosing interval [29–32]. The results presented in this study support further investigation of imlunestrant as the ET backbone aimed to overcome the limitations of fulvestrant and provide a combination regimen that includes HER2 + targeted therapy.

Combining the ET backbone with a CDK4/6i is currently a preferred SOC [33, 34] for the treatment of ER+/HER2- ABC [35]. The recently published EMBER-3 study (NCT04975308) demonstrated a notable PFS of 9.4 months with the combination of imlunestrant + abemaciclib in patients with ER+/HER2- ABC previously treated with an aromatase inhibitor [36]. Furthermore, data from the EMBER trial combining imlunestrant + abemaciclib to targeted therapy supports combination therapy by demonstrating a manageable safety profile and signals of efficacy [18, 36].

Given the aggressive nature of HER2 + disease and the evolution of targeted therapies, it is important to contextualize the role of HER2 blockade in improving outcomes. Tumors with HER2 overexpression (both ER + and ER-) have historically represented a clinically aggressive subtype of breast cancer. The introduction of HER2 targeted therapies has transformed the treatment landscape, significantly improving the prognosis of HER2 + breast cancer [37].

The PERTAIN trial demonstrated that dual HER2 blockade with pertuzumab and trastuzumab, combined with an aromatase inhibitor, improves efficacy in ER+, HER2 + metastatic breast cancer. This combination improved progression-free survival (PFS), with a median of 18.9 months (95% CI: 14.1–27.7), compared to 15.8 months (95% CI: 11.0–18.6) with trastuzumab plus an aromatase inhibitor alone [38].

The efficacy of adding the CDK4/6 inhibitor palbociclib to HER2 targeted therapy was assessed in two trials involving patients with HR+/HER2 + metastatic BC. In the TLP trial (NCT03054363), 42 patients received a combination of HER2-targeted therapy (tucatinib), an aromatase inhibitor (letrozole), and palbociclib, resulting in a median PFS of 8.4 months, and CBR of 70.4% [39].

More recently, the AFT-38 PATINA trial (NCT02947685) evaluated the addition of palbociclib to HER2-targeted therapy (trastuzumab ± pertuzumab) and ET following induction therapy in patients with ER + HER2 + MBC. This addition significantly extended PFS, with a median PFS of 44.3 months, compared to 29.1 months in the control arm (*p* = 0.0074) and a CBR of 89.3% [9].

The crosstalk between ER and HER2 receptors allows compensatory escape pathways and dual blockade targeting both pathways may overcome this resistance [3]. As ET combined with HER2 + targeted agents delivered similar efficacy as with trastuzumab + chemotherapy, further studies to evaluate imlunestrant as a de-escalation strategy that allows chemotherapy omission are warranted [40].

HER2-targeted therapy can also delay brain metastases, an important consideration given that HER2 + disease has higher rates of CNS disease [41]. In mouse tumor orthotopic models, imlunestrant crossed the blood-brain barrier and improved survival [19]. Further evaluation into whether imlunestrant + HER2-targeted therapy can delay or prevent brain metastases is warranted. EMBER-3 included patients with controlled brain metastases and data suggests that imlunestrant may reduce CNS progression in patients with stable brain metastasis [36]. These studies support imlunestrant as a potential ET backbone with HER2-targeted therapy for ER+/HER2 + ABC.

Imlunestrant combined with targeted HER2 therapy (trastuzumab, pertuzumab) and a CDK4/6i (abemaciclib) exhibited antitumor activity in patients with ER-+/HER2 + ABC. Furthermore, imlunestrant combined with HER2-targeted therapy revealed a favorable safety profile, with manageable, mainly low-grade TEAEs, and low rates of dose modifications in groups A and C. However, we did observe somewhat higher incidences of TEAEs, incidence of grade ≥ 3 TEAEs, and rate of dose modifications with group B. These were consistent with other abemaciclib combinations [18].

The monarcHER (NCT02675231) and PATRICIA (NCT02448420) trials revealed a synergistic benefit in co-inhibition of CDK4/6 and HER2 in ER+/HER2 + ABC. In these studies, the ER+/HER2 + cohorts treated with fulvestrant + trastuzumab + CDK4/6i experienced high incidence of grade ≥ 3 TEAEs (monarcHER: 63% [*n* = 49]; PATRICIA: 86% [*n* = 24]) [14, 42]. In this EMBER study, fewer (52%) patients treated with imlunestrant + trastuzumab + abemaciclib experienced grade ≥ 3 events. While cross-trial comparisons are limited, this observation suggests that this combination may be associated with a manageable safety profile in the context of HER2-targeted agents.

The most frequently altered genes in this study were *ERBB2*, *TP53*, *PIK3CA*, *GATA3*, *ESR1* and *CCND1*, aligning with previous studies [43–45]. These genetic alterations compare to those previously reported for HR+/HER2 + ABC using the MSK-IMPACT assay in 109 patients, reflecting high consistency of the population’s genetic profile and emphasizing the reliability of these genetic alterations as significant drivers of ER+/HER2 + BC [46].

The prevalence of *ESR1* mutations detected in EMBER was higher though likely related to the sample type. EMBER tested blood (ctDNA) whereas tissue samples were used in comparable studies. Furthermore, prior therapy differences can contribute to variances. The higher prevalence of *ESR1* mutations (Table 1) presumably reflects that more than half of the EMBER patients had prior aromatase inhibitors which are associated with acquired *ESR1* mutations [47, 48]. The relatively low ctDNA detection of *ERBB2* amplification (57%) likely relates to insufficient DNA shedding to enable detection.

Our study is limited by the small sample size in all cohorts, especially the maintenance cohort. Results must be interpreted with caution, particularly in patients with an *ESR1* mutation. Further, maintenance patients were not exposed to ET and were at maximal response prior to study entry. Ultimately, these data can be considered hypothesis-generating. Future randomized studies are warranted to confirm these findings and explore the role of imlunestrant in de-escalation strategies and CNS disease management.

In summary, our results suggest a potential benefit for the combination of imlunestrant and HER2 targeted therapy, as a nonchemotherapeutic approach in ER + HER2 + ABC, supporting further investigation.

## Conclusion

Imlunestrant treatment in combination with HER2-targeted therapy revealed a manageable safety profile with evidence of preliminary efficacy in patients with ER+/HER2 + ABC. Imlunestrant, when combined with other agents, demonstrated fewer, manageable, high-grade events compared to recently published HER2-targeted combinations with abemaciclib [14, 42]. Further studies are warranted, particularly to evaluate the potential of delaying brain metastasis. Finally, imlunestrant +/- abemaciclib could be evaluated as the ET backbone in the maintenance setting following first-line taxane-based therapy for ER+/HER2 + ABC.

## Supplementary Information

Below is the link to the electronic supplementary material.

Supplementary Material 1

## Data Availability

No datasets were generated or analysed during the current study. Eli Lilly and Company provides access to all individual participant data collected during the trial, after anonymization, with the exception of pharmacokinetic or genetic data. Data are available to request 6 months after the indication studied has been approved in the United States and European Union and after primary publication acceptance, whichever is later. No expiration date of data requests is currently set once data are made available. Access is provided after a proposal has been approved by an independent review committee identified for this purpose and after receipt of a signed data sharing agreement. Data and documents, including the study protocol, statistical analysis plan, clinical study report, blank or annotated case report forms, will be provided in a secure data sharing environment. For details on submitting a request, see the instructions provided at www.vivli.org.

## Abbreviations

ABC: Advanced Breast Cancer
AE: Adverse Event
BC: Breast Cancer
CBR: Clinical Benefit Rate
CDK4/6: Cyclin-Dependent Kinase 4 and 6
CI: Confidence Interval
CR: Complete Response
ctDNA: Circulating Tumor DNA
DCR: Disease Control Rate
DDI: Drug-Drug Interaction
EBC: Early Breast Cancer
ECOG PS: Eastern Cooperative Oncology Group Performance Status
EEC: Endometrioid Endometrial Cancer
ER: Estrogen Receptor
ET: Endocrine Therapy
FDA: Food and Drug Administration
HER2: Human Epidermal Growth Factor Receptor 2
HR+: Hormone Receptor-Positive
LC-MS/MS: Liquid Chromatography-Mass Spectrometry
MUGA: Multigated Acquisition
ORR: Objective Response Rate
PFS: Progression-Free Survival
PK: Pharmacokinetics
PR: Partial Response
RP2D: Recommended Phase 2 Dose
SAE: Serious Adverse Event
SERD: Selective Estrogen Receptor Degrader
SOC: Standard of Care
TEAE: Treatment-Emergent Adverse Event
TRAE: Treatment-Related Adverse Event
