# Supplementary material for: Imlunestrant, an oral selective estrogen receptor degrader, in combination with HER2 directed therapy, with or without abemaciclib, in ER-positive, HER2-positive advanced breast cancer: results from the phase 1a/1b EMBER study
Source: Breast Cancer Res. 2025 Dec 21;28:18. doi: 10.1186/s13058-025-02168-6 (PMC12829183; doi:10.1186/s13058-025-02168-6)
Supplement: Supplementary file 1 — Supplementary Material 1 [file 13058_2025_2168_MOESM1_ESM.docx]

***Supplementary Information***

***Table S1. SAEs***

|  | Imlunestrant + trastuzumab | Imlunestrant + trastuzumab + abemaciclib | Imlunestrant + trastuzumab + pertuzumab |
| --- | --- | --- | --- |
| Parameters, n (%) | n=18 | n=21 | n=6 |
| Patients with ≥ 1 SAE | 4 (22) | 4 (19) | 0 |
| Pneumonitis | 0 | 2 (10) | 0 |
| Cholecystitis | 1 (6) | 0 | 0 |
| Clostridium difficile infection | 0 | 1 (5) | 0 |
| Jugular vein thrombosis | 1 (6) | 0 | 0 |
| Mastitis | 1 (6) | 0 | 0 |
| Pain | 0 | 0 | 0 |
| Pneumothorax | 0 | 1 (5) | 0 |
| Sepsis | 1 (6) | 0 | 0 |
| Septic Shock | 0 | 1 (5) | 0 |
| Pneumonia | 1 (6) | 0 | 0 |

***Table S2. Efficacy parameters in patients***

|  | Imlunestrant + trastuzumab | Imlunestrant + trastuzumab + abemaciclib | Imlunestrant + trastuzumab + pertuzumab |
| --- | --- | --- | --- |
|  | n=18 | n=21 | n=6 |
| CBR, n (%) | 8 (44) | 10 (48) | 6 (100) |
| DCR, n (%) | 11 (61) | 13 (62) | 6 (100) |
| PFS |  |  |  |
| Median, months (95% CI) | 5.3 (1.9, 6.6) | 6.7 (2.7, 12.4) | 15.8 (8.3, NE) |
| 6-month PFS rate, % (95% CI) | 38.9 (17.5, 60) | 50 (25.9, 70.1) | 100 (100, 100) |
| 12-month PFS rate, % (95% CI) | 16.7 (4.1, 36.5) | 33 (13.7, 54.5) | 66.7 (19.5, 90.4) |
| 18-month PFS rate, % (95% CI) | NA | 25 (7.5, 47.6) | 0 (NE, NE) |
| Median treatment duration, months (range) | 5.8 (1.4-18.9) | 4.2 (0.3-24.6) | 11.3 (8.3-16.4) |
| Median TTR, months (range) | 5.8 (5.7-5.7) | 1.8 (1.5-3.7) | NA |
| ORR evaluable population, n ^a^ | n=14 | n=20 | n=3 |
| ORR, n (%) | 1 (7) | 5 (25) | 1 (33) |
| Complete response | 0 | 0 | 0 |
| Partial response | 1 (7) | 5 (25) | 1 (33) |
| Stable disease | 6 (43) | 7 (35) | 2 (67) |
| Persistent for ≥ 24 weeks | 3 (21) | 4 (20) | 2 (67) |
| Progressive disease | 7 (50) | 4 (20) | 0 |
| Non evaluable | 0 | 4 (20) | 0 |

Abbreviations: CBR, clinical benefit rate (best overall response of complete response and partial response or stable disease for ≥24 weeks); CI, confidence interval; ER+, estrogen receptor-positive; HER2+, human epidermal growth factor receptor 2 positive; NA, not available; n, number of patients in the specific category; ORR, overall response rate (best overall response of complete response and partial response); PFS, progression-free survival; TTR, time to response.

^a^ORR was evaluable for patients who had measurable disease at baseline.
